# In Vitro Model of Human Skeletal Muscle Tissue for the Study of Resident Macrophages and Stem Cells

**DOI:** 10.3390/biology11060936

**Published:** 2022-06-19

**Authors:** Dandan Hao, Nils Becker, Eva Mückter, Aline Müller, Miguel Pishnamaz, Leo Cornelis Bollheimer, Frank Hildebrand, Mahtab Nourbakhsh

**Affiliations:** 1 Clinic for Geriatric Medicine, RWTH Aachen University Hospital, 52074 Aachen, Germany; dhao@ukaachen.de (D.H.); emueckter@ukaachen.de (E.M.); almueller@ukaachen.de (A.M.); cbollheimer@ukaachen.de (L.C.B.); 2 Clinic for Orthopedics, Trauma, and Reconstructive Surgery, RWTH Aachen University Hospital, 52074 Aachen, Germany; nibecker@ukaachen.de (N.B.); mpishnamaz@ukaachen.de (M.P.); fhildebrand@ukaachen.de (F.H.)

**Keywords:** human, in vitro model, aging, skeletal muscle, tissue, myofibers, myofibrils, satellite cells, myoblasts, resident macrophages, actin, desmin, immunohistology

## Abstract

**Simple Summary:**

The skeletal muscle of younger adults has a remarkable regenerative capacity, which substantially declines with age. Despite many interspecies differences, animals have been used to study new treatments to promote muscle regeneration in humans. This study reports a novel human experimental model using human skeletal muscle tissue of older adults that was extracted during surgical procedures. We describe an optimal procedure for maintaining human skeletal muscle tissue under experimental conditions for 11 days. This experimental model allows the investigation of resident macrophages and stem cells, which mediate muscle regeneration.

**Abstract:**

Findings from studies of muscle regeneration can significantly contribute to the treatment of age-related loss of skeletal muscle mass, which may predispose older adults to severe morbidities. We established a human experimental model using excised skeletal muscle tissues from reconstructive surgeries in eight older adults. Muscle samples from each participant were preserved immediately or maintained in agarose medium for the following 5, 9, or 11 days. Immunofluorescence analyses of the structural proteins, actin and desmin, confirmed the integrity of muscle fibers over 11 days of maintenance. Similarly, the numbers of CD80-positive M1 and CD163-positive M2 macrophages were stable over 11 days in vitro. However, the numbers of PAX7-positive satellite cells and MYOD-positive myoblasts changed in opposite ways, suggesting that satellite cells partially differentiated in vitro. Further experiments revealed that stimulation with unsaturated fatty acid C18[2]c (linoleic acid) increased resident M1 macrophages and satellite cells specifically. Thus, the use of human skeletal muscle tissue in vitro provides a direct experimental approach to study the regulation of muscle tissue regeneration by macrophages and stem cells and their responses to therapeutic compounds.

## 1. Introduction

Skeletal muscle, which makes up approximately 40% of the total weight of the human body, plays a pivotal role in the regulation of homeostasis and metabolism and enables body movement and respiration [1]. Aging is accompanied by a successive decline in skeletal muscle mass and function, which has deleterious impacts on metabolism, the immune system, and mobility. This age-related decline in muscle mass has been attributed to decreases in the number and size of muscle fibers [2]. Skeletal muscle is mainly composed of slow-twitch fibers (Type I) and fast-twitch fibers (Type II), which includes the subtypes IIA, IIB, and IIX [1]. Previous studies reported that age-related muscle mass loss is mainly attributed to a reduction in type II muscle fiber size [2]. The regeneration of skeletal muscle fibers heavily relies on the coordinated actions of resident macrophages and quiescent stem cells [3]. Recent findings revealed the modulating function of resident macrophages in both the regeneration and degeneration of skeletal muscle fibers [3,4]. Muscle stem cells were named satellite cells due to their location between the basal lamina and the sarcolemma of the myofibers. Upon activation, quiescent satellite cells divide both symmetrically and asymmetrically to generate activated satellite cells and self-renewing satellite cell stem cells, respectively [5]. After a few rounds of cell division, activated satellite cells (myoblasts) exit their cell cycles and give rise to myocytes, which fuse together to form multinucleated myotubes. [5,6]. Dysregulation of any step within this strictly regulated process can result in the successive loss of muscle mass and strength, which results in debilitating conditions [7]. Satellite cells were previously characterized by positive expression of the PAX7 transcription factor, which is abolished during myogenic differentiation [8]. Concomitantly, expression of the early myogenic transcription factor MYOD increases, so MYOD is commonly used to detect differentiated skeletal myoblasts [9].

Obesity increases the risk of age-related decline in muscle mass, suggesting a direct link between the excessive accumulation of fat and fatty acids in muscle tissue and muscle degeneration [10,11,12]. Animal studies demonstrated that specific species of fatty acids can directly induce inflammatory gene expression in the skeletal muscle tissue of obese rats [11]. Recently, we reported that 18-carbon fatty acid with two unsaturated double bonds in cis configuration (C18[2]c) modulates the expression of eight chemokine genes (IL6, IL1RA, IL4, LIF, CXCL8, CXCL1, CXCL12, and CCL2) in primary human skeletal muscle myoblasts [13]. This led to the assumption that fatty-acid-induced chemokines may trigger the activation of resident macrophages in human muscle tissue.

In skeletal muscle tissue, macrophages represent the most abundant class of immune cells. They contribute to muscle regeneration by cleaning tissue debris, regulating proinflammatory or anti-inflammatory responses, and activating satellite cell proliferation and differentiation [4,14]. Resident macrophages also undergo a series of strictly regulated steps to ensure efficient tissue homeostasis. In general, macrophages comprise two functionally distinct populations, the proinflammatory M1 subtype and the anti-inflammatory M2 subtype [15]. However, a more complex system of macrophage subtypes may exist and is the subject of ongoing functional studies [16]. The use of numerous different markers from human and animal models or from blood-derived and in vitro-differentiated precursor macrophages has complicated the characterization of macrophages [16,17,18,19]. Nonetheless, the current classification of M1 and M2 macrophages provides a useful guide to study the regulatory mechanisms of macrophage polarization and the inflammatory status of human tissue.

Animal models have been generally utilized to replicate and study various aspects of human skeletal muscle disorders [20,21]. A few in vitro models of human skeletal muscle have been developed using isolated primary myoblasts, myotubes, or immortalized cell lines [22,23,24]. However, none of these models represent the intrinsic complexity of the native human skeletal muscle tissue, which is composed of many different types of cells and structural components. Here, we report the development of a human skeletal muscle tissue model that can be used to study the interaction of skeletal muscle stem cells and macrophages in their natural environment and to study new regenerative treatments. Compared to monolayer cultured cells, three-dimensional skeletal muscle tissue better mimics the natural tissue environment and allows satellite cells and myoblasts to maintain an extracellular matrix in three dimensions and to interact with resident macrophages through signaling factors.

## 2. Materials and Methods

### 2.1. Tissue Specimens

Human skeletal muscle samples were obtained from eight geriatric donors undergoing reconstructive surgery at RWTH Aachen University Hospital, Aachen, Germany, after November 2020 (Table 1). Ethical approval from the Medical Ethics Committee of RWTH Aachen (No. EK206/09) and patient consent were obtained before the surgeries.

### 2.2. Maintenance and Stimulation of Human Skeletal Muscle Tissue

Tissue samples from each participant were sectioned into several serial sections of ≈5.3 mm^3^. One section was immediately preserved in a 4% formaldehyde solution (Otto Fischar GmbH, Saarbrucken, Germany) at 4 ℃ (D0). The remaining sections were separately embedded in a polysaccharide polymer (agarose). Briefly, the embedding procedure consisted of 2 consecutive steps: sealing using superficial dehydration by airflow; and embedding in 1% ultrapure polysaccharide polymer Low Melt-Agarose (Carl Roth GmbH, Karlsruhe, Germany) in DMEM cell culture medium (Biological industries, Kibbutz Beit-Haemek, Israel), supplemented with 10% fetal bovine serum (PAN Biotech GmbH, Aidenbach, Germany) along with penicillin and streptomycin, each at 100 U/mL (PAN Biotech GmbH, Aidenbach, Germany). For stimulation, analytical-grade fatty acid C18[2]c was obtained from Biotrend Chemikalien GmbH, Cologne, Germany (#1024), and conjugated with bovine serum albumin (BSA) at a 1:2.5 ratio, as described previously [13]. C18[2]c solution was added to the agarose medium at a final concentration of 50 µm. The units of embedded tissues were covered with 0.5 mL of DMEM/F-12 medium (Gibco Life Technologies), supplemented with 10% fetal bovine serum along with penicillin and streptomycin, each at 100 U/mL (PAN Biotech GmbH), and incubated at 37 °C with 5% CO_2_. The medium was replaced every five days. After maintenance for the indicated duration (5, 9, or 11 days), the tissue sections were carefully captured and preserved in a 4% formaldehyde solution (Otto Fischar GmbH) for 24 h. 

### 2.3. Hematoxylin and Eosin (HE) Staining

The tissue samples were dehydrated, embedded in paraffin, and sectioned into 2 μm sections using a SLIDE4003E microtome (pfm Medical, Cologne, Germany). The sections were fixed on adhesive microscope slides, deparaffinized, and stained using an automated slide-staining station (Gemini, Thermo Fisher, Waltham, MA, USA). The slides were stained in hematoxylin for 5–10 min; rinsed in warm water for 10 min; stained in 0.3% eosin for 5 min; rinsed again with tap water; and successively dehydrated in 70%, 96%, and 100% ethanol. Then, the slides were treated with xylene and sealed with glass coverslips. 

### 2.4. Immunofluorescence and DAPI Staining

Five-micrometer sections of embedded tissue samples were deparaffinized, heated in citrate buffer (pH 6.0) for 30 min, cooled, and left in distilled water. Before staining, the sections were rinsed twice with 0.1% Tween 20 (9127.1, Carl Roth GmbH, Karlsruhe, Germany) in PBS. For PAX7 and MYOD staining, slides were permeabilized in 0.1% Triton X-100 (T8787, Sigma-Aldrich Chemic, Steinheim, Germany) for 10 min and blocked with UltraCruz Blocking Reagent for 60 min. For CD80 and CD163 staining, the slides were blocked using 10% BSA in PBS for 60 min. The slides were incubated with the following antibodies overnight at 4 ℃: rabbit anti-human CD80 (ab134120, Abcam, Cambridge, UK) diluted 1:1000 in 3% BSA, mouse anti-human CD163 (ab156769, Abcam, Cambridge, UK) diluted 1:200 in 3% BSA, mouse anti-human Pax-7 (sc-81975, Santa Cruz Biotechnology, Dallas, TX, USA) diluted 1:100 in UltraCruz Blocking Reagent, or mouse anti-human MYOD (sc-377460 AF488, Santa Cruz Biotechnology) 1:100 in UltraCruz Blocking Reagent (Santa Cruz Biotechnology). For actin and desmin staining, two-micrometer tissue sections were deparaffinized and treated as described above. Then, the sections were permeabilized for 10 min using 0.1% Triton X-100 (T8787, Sigma-Aldrich Chemic, Steinheim, Germany) in PBS and blocked with 2.5% horse serum for 60 min. The slides were then incubated with the following primary antibodies overnight at 4 ℃: mouse anti-human skeletal muscle actin antibody (MA5-12542, Thermo Fisher Scientific, Waltham, MA, USA) diluted 1:200 in 2.5% horse serum or rabbit anti-human skeletal muscle desmin antibody (ab227651, Abcam, Cambridge, UK) diluted 1:200 in 2.5% horse serum. After primary staining, the slides were treated with two reagents from the VectaFluor Amplified Kit (DK2488 or DK1594, Vector Laboratories, Burlingame, CA, USA). After staining, the slides were sealed with glass coverslips in SlowFade Gold Antifade Mountant with DAPI (Thermo Fisher Scientific, Waltham, MA, USA).

### 2.5. Microscopy and Imaging

Following HE or immunofluorescence staining, images were captured using an automated microscope (DM6000B, Leica Microsystems, Wetzlar, Germany) with a 340–380 nm filter for DAPI; a 450–490 nm filter for PAX7, MYOD, actin or CD163; and a 590 nm filter for desmin or CD80. DISKUS software (Leica) was used to process and merge the images. The mean number of positive cells in each sample was obtained from at least two randomly selected fields of 0.24 mm^2^ within cross-sectional areas of muscle fibers. The fields containing adipose tissue, glands, or vessels were strictly excluded.

### 2.6. Statistical Analyses

All results are reported as the mean ± standard deviation (SD). Statistical analyses were performed with SPSS 18.0. The results were compared using a *t*-test. Differences with a *p* value ≤ 0.01 were considered statistically significant.

## 3. Results

The study of skeletal muscle regeneration in older adults is in high demand in geriatric medicine. In the current study, we included eight older adults undergoing reconstructive surgical procedures. All surgeries involved an obligatory macroscopic excision of skeletal muscle tissue to restore skeletal shape and function. All participants gave their informed consent to donate their disposed tissue. The participants’ characteristics are summarized in Table 1. In general, female participants were more willing to donate than male patients. Moreover, the share of female patients in our hospital significantly increases with age. Thus, only one male participant was included in this study.

### 3.1. The Integrity of Human Skeletal Muscle Tissue under Experimental Conditions In Vitro

**Figure 1:**
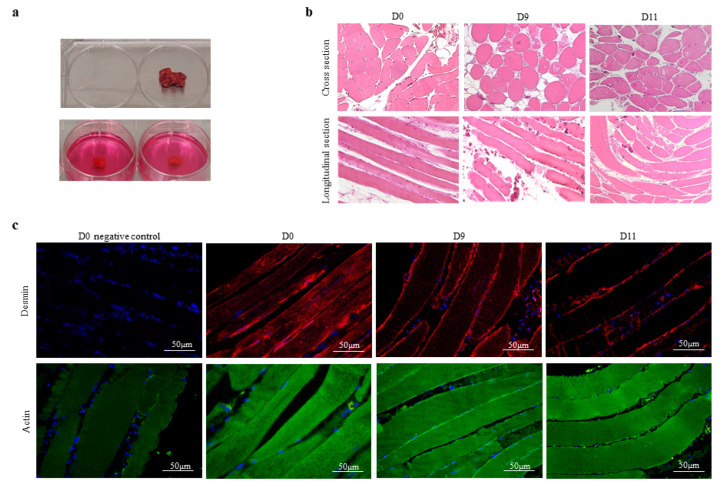
Human skeletal muscle tissue can be maintained under in vitro conditions. (**a**) A skeletal muscle tissue sample from a single participant (upper panel) was sectioned into multiple units that were embedded in agarose medium (lower panel). (**b**) The images from a single participant are presented and are representative of an additional seven participants. The morphological characteristics of tissue samples from the vastus lateralis muscle of individual participants were analyzed in longitudinal and cross sections by HE staining immediately after surgery (D0) or after 9 or 11 days of maintenance in vitro (D9 or D11, respectively). (**c**) Parallel sets of tissue sections from the gluteus medius muscle at D0, D9, and D11 were analyzed, and the morphological integrity of muscle fibers was compared using antibodies against desmin (upper panel) or actin (lower panel) for immunofluorescence staining, with DAPI used as a counterstain. Additional sections were analyzed without the addition of desmin or actin antibodies as a negative control (D0 negative control). Images of tissue from a single participant are shown and are representative of the other seven participants. The white scale bars represent 50 μm.

Skeletal muscle tissue samples were sectioned into four serial sections immediately after surgery. One section was directly preserved and served as the control (D0). The other three sections were embedded in individual wells of a six-well culture plate (Figure 1a). After being maintained in vitro for 5, 9 or 11 days (D5, D9 or D11, respectively), the tissue sections were preserved and prepared for histological assessment. Next, the tissue sections were subjected to HE staining to study their general arrangement and the distribution of muscle fibers and to obtain a general overview of cross-sectional and longitudinal tissue sections (Figure 1b). All sections contained muscle fibers of different lengths (lower panel) as well as adipose and connective tissues (upper panel). Longitudinal tissue sections revealed that short muscle fibers remained intact in the sections while long muscle fibers were truncated by the excisions. As expected, there were individual differences in the proportions of adipocytes and connective structures between the participants’ muscle tissues. We then focused on muscle fibers and examined different qualitative and quantitative parameters, such as the shape and size of the muscle fibers, the number and location of nuclei, and the presence of necrosis in the samples, at D0, D5, D9, and D11 for each participant. In all samples, the nuclei were peripheral, and the sarcoplasm appeared uniform and unfragmented over time. Most importantly, the maintenance conditions did not have a significant effect on the organization of muscle fibers within the tissue samples from individual participants (Figure 1b). To further examine the integrity and structure of the muscle fibers over the maintenance period, all tissue sections were simultaneously subjected to immunofluorescence staining using specific antibodies against two major muscle filament proteins, actin and desmin [25,26]. In addition, DAPI was utilized as a nuclear counterstain to exclude alterations in nuclear abundance or appearance across the tissue [27]. In all samples, we observed a low, constant ratio of 0.1 ± 0.04% of fragmented, condensed, or deformed nuclei, independent of donor characteristics or maintenance duration. As shown in Figure 1c, the desmin signal strongly accumulated in the sarcolemma but was less abundant in myofibrils at D0. This pattern was more apparent at D9 and D11, which is most likely due to the disparate spatial distribution of desmin filaments. Desmin links myofibril bundles on specialized sites in the sarcolemma. Depending on the location of tissue slice, desmin signals may appear stronger in the sarcolemma. Importantly, we observed no alterations in the density or organization of actin filaments over 11 days (D11) of maintenance in vitro. The sarcolemma and myofibrils contained equal amounts of actin in all samples. Moreover, neither the number nor the appearance of nuclei across the tissue sections was significantly different. Thus, the data strongly suggested that vital human skeletal muscle tissue can be maintained under native conditions in vitro for at least 11 days.

### 3.2. The In Vitro Model of Human Skeletal Muscle Maintains the Polarization Profile of Resident Macrophages

**Figure 2:**
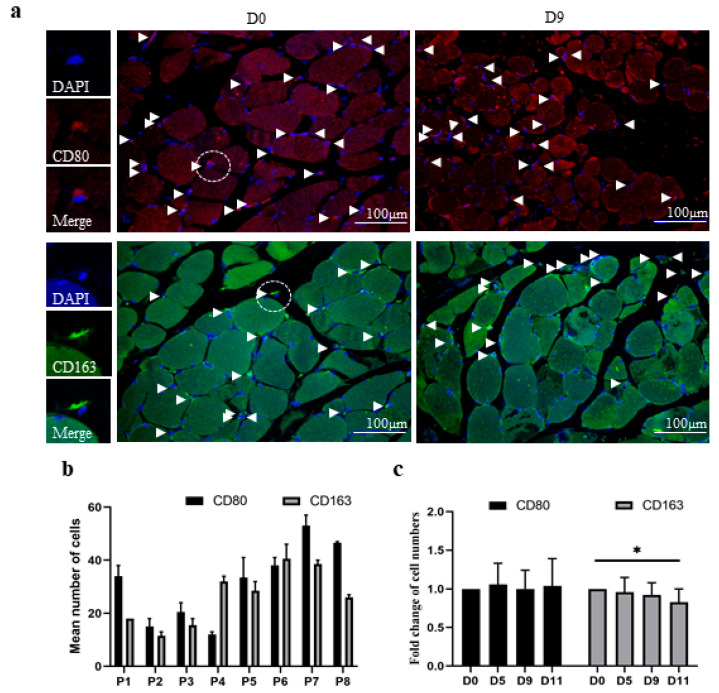
Resident macrophages in human skeletal muscle tissue maintained in vitro for 11 days. Skeletal muscle tissue sections were analyzed for the presence of M1 (CD80-positive) or M2 (CD163-positive) macrophages using immunofluorescence and DAPI staining. (**a**) The images are representative of a total of 32 different sections from 8 participants before (D0) or after 5 (D5), 9 (D9), or 11 (D11) days of maintenance in vitro. Sections from a single participant at D0 and D9 are presented and labeled at the top of each image. White arrowheads indicate positive cells. The white scale bars represent 100 μm. Left panels present fluorescence images at higher magnification of single M1 macrophages (red), M2 macrophages (green), DAPI (blue), and merged signals, as depicted. The mean number of positive cells was obtained from two randomly selected fields of 0.24 mm^2^ within cross-sectional areas of muscle fibers. (**b**) The diagram shows the mean numbers of CD80-positive (black bars) and CD163-positive (gray bars) macrophages in 0.24 mm^2^ of muscle fibers from individual participants (P1–P8) at D0 (Supplementary Table S1). (**c**) The diagram shows the mean fold change, which was determined by normalizing the number of positive cells in samples from each participant at D0, D5, D9, and D11 to that at D0. Thus, D0 was set to one for every participant and for the mean change at D0 (Supplementary Table S2). *p* ≤ 0.01 (*).

To assess the polarization profile of resident macrophages in muscle tissue sections, we utilized immunofluorescent staining protocols to exclusively detect M1 (CD80^+^/DAPI^+^) or M2 (CD163^+^/DAPI^+^) macrophages. As shown in representative fluorescence images from a single donor at D0 and D9, positive cells were labeled and counted in equal fields of muscle fibers within the corresponding tissue sections (Figure 2a). M1 and M2 macrophages were found in the perimysium and exhibited a similar lanceolate shape, with the nucleus located toward the rear of the cell, which has been previously described for migrating cells (Figure 2a). We determined and compared the numbers of M1 and M2 macrophages in the muscle tissue samples from all participants (Figure 2b,c). We observed that the numbers of M1 and M2 macrophages at D0 varied among the participants (Figure 2b).

Furthermore, the individual ratios of M1 to M2 macrophage subtypes also significantly differed. For instance, the muscle tissue of participant one contained more M1 macrophages than M2 macrophages, whereas that of participant four contained more M2 macrophages than M1 macrophages (Figure 2b). Next, we determined the relative changes in macrophage numbers during in vitro maintenance for each individual participant (Figure 2c). Therefore, the number of M1 macrophages or M2 macrophages was compared to the corresponding number at D0 to obtain a relative fold change, which was set to one for all participants at D0. The results revealed an unchanged number of M1 macrophages and a negligible decline in M2 macrophages over 11 days of in vitro maintenance.

### 3.3. The In Vitro Model of Human Skeletal Muscle Maintains the Number and Differentiation of Skeletal Muscle Stem Cells

**Figure 3:**
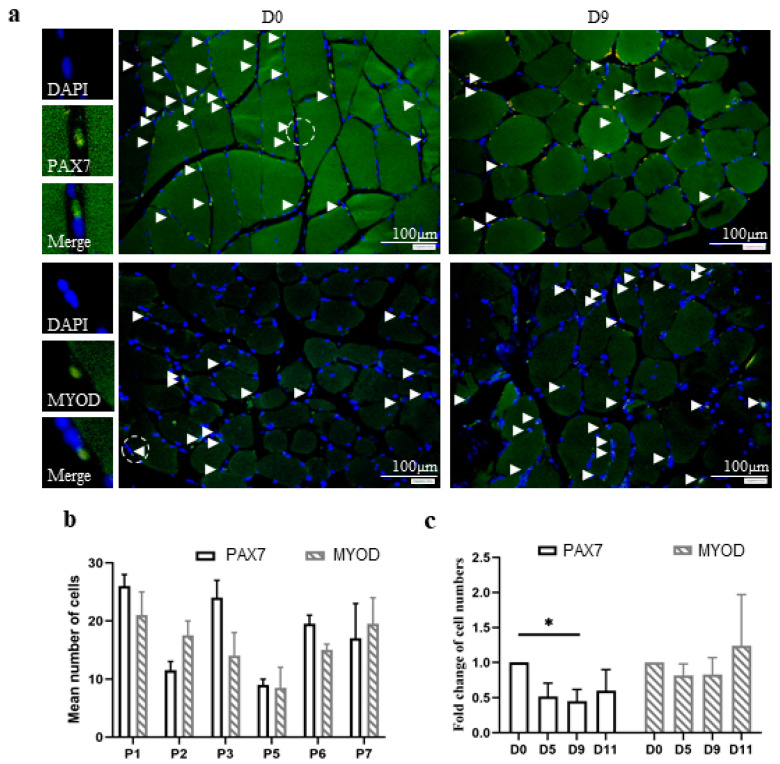
Stem cells in human skeletal muscle tissue were maintained in vitro for 11 days. Skeletal muscle tissue sections were analyzed for the presence of satellite cells (PAX7-positive) or myoblasts (MYOD-positive) using immunofluorescence and DAPI staining. (**a**) The images are representative of all sections from 6 participants before (D0) or after 5 (D5), 9 (D9), or 11 (D11) days of maintenance in vitro. Sections from a single participant at D0 and D9 are presented and labeled at the top of each image. White arrowheads indicate the positive cells. The white scale bars represent 100 μm. Left panels present fluorescence images at higher magnification of a single satellite cell or myoblast (green), DAPI (blue), and merged signals, as depicted. The mean numbers of positive cells from two randomly selected fields of 0.24 mm^2^ within cross-sectional areas of muscle fibers were obtained. (**b**) The diagram shows the mean numbers of PAX7-positive satellite cells (white bars) and MYOD-positive myoblasts (hatched bars) in 0.24 mm^2^ of muscle fibers from individual participants as designated at D0 (Supplementary Table S3). (**c**) The diagram shows the mean fold change, which was determined by normalizing the number of PAX7-positive (white bars) or MYOD-positive (hatched bars) cells in the samples from each participant at D0, D5, D9, and D11 to that at D0. Thus, D0 was set to one for every single participant and for the mean change at D0 (Supplementary Table S4). *p* ≤ 0.01 (*).

Stem cells play a pivotal role in the regeneration of skeletal muscle tissue. We used antibodies against two well-established markers, PAX7 and MYOD, to identify satellite cells and myoblasts, respectively, in human skeletal muscle tissue samples. PAX7 and MYOD are transcription factors and are predominantly located in the nucleus. Thus, their fluorescence signals often overlapped or exceeded the underlying DAPI signals. Positive cells were identified by overlapping blue and green fluorescence signals and counted in equal fields of muscle fibers as described above (Figure 3a). The examination of the tissue samples from different participants at D0 revealed that the majority contained comparable numbers of satellite cells and myoblasts (Figure 3b). 

As expected, the individual ratios of satellite cells to myoblasts were not significantly different. We determined the relative fold changes in satellite cell and myoblast numbers by comparing the numbers with those at D0 (Figure 3c). Thus, the relative change at D0 was set to one. The results indicated that the number of satellite cells successively decreased over 11 days of maintenance in vitro. However, the number of myoblasts was slightly increased. Interestingly, the sum of satellite cells and myoblasts indicated nearly no relevant change (Supplementary Figure S1). We assumed that a small proportion of satellite cells may have differentiated into myoblasts during maintenance in vitro.

### 3.4. Unsaturated C18 Fatty Acid Activates M1 Macrophages and Stem Cells in the Human Skeletal Muscle Model

**Figure 4:**
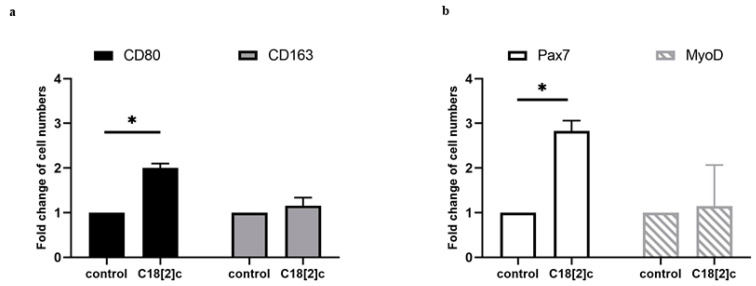
M1 macrophages and satellite cells were increased in human skeletal muscle tissue in response to unsaturated fatty acid C18[2]c. Skeletal muscle tissue sections from donor 6 were stimulated with C18[2]c or left untreated for 9 days (control) and analyzed for the presence of M1 (CD80-positive) or M2 (CD163-positive) macrophages, satellite cells (PAX7-positive), or myoblasts (MYOD-positive) using immunofluorescence and DAPI staining. (**a**) The diagram shows the mean fold change of M1 (CD80-positive) (black bars) and M2 (CD163-positive) macrophages (gray bars) in 0.24 mm^2^ of muscle fibers, which was determined by normalizing the number of cells in the stimulated sample to that of the unstimulated sample. (**b**) The diagram shows the mean fold change of PAX7-positive satellite cells (white bars) and MYOD-positive myoblasts (hatched bars) in 0.24 mm^2^ of muscle fibers, which was determined by normalizing the number of cells in the stimulated sample to that of the unstimulated sample. Thus, the controls were set to one. *p* ≤ 0.01 (*).

Previously, we reported that unsaturated fatty acid C18[2]c stimulates the expression of inflammatory proteins in primary human skeletal myoblasts [13]. This led to the assumption that the elevated inflammatory proteins may potentially activate muscle-tissue-resident macrophages. We were able to examine this hypothesis in participant six, who provided additional sections of the vastus lateralis muscle. Tissue samples were maintained with C18[2]c/BSA solution or a fatty-acid-free BSA solution for nine days in vitro. We determined the relative changes in macrophage and stem cell numbers as described above (Figure 2c and Figure 3c). The data revealed that only CD80-positive M1 macrophages and PAX7-positive satellite cells were significantly increased in response to C18[2]c stimulation (Figure 4a,b). Together, our data indicated the viability of the human skeletal muscle model for the experimental study of macrophage and satellite cells in response to stimuli. 

## 4. Discussion

The age-related decline in muscle regeneration results in impaired mobility, increased injury risk, and dependency, which pose great challenges to the healthcare system. A better understanding of the underlying mechanisms will assist in the development of new therapies for the growing population of older adults. Our study presents a new in vitro human model for the further investigation of the regulatory crosstalk between resident stem cells and macrophages within the native three-dimensional microenvironment of skeletal muscle tissue. The established embedding procedure in agarose medium maintained muscle tissue integrity and the macrophage profile under controlled conditions, allowing free diffusion of nutrients and satellite cell differentiation. Furthermore, the resident macrophages and stem cells responded to external stimuli under in vitro conditions. These features suggest the capability of the human skeletal muscle tissue model to reduce animal experiments and may advance its acceptance in the field of aging research.

The experiments in this report demonstrate the viability of the human skeletal muscle model over 11 days in vitro. In preliminary experiments, however, we also maintained parallel samples for 13 days and observed some modifications to the actin structure (data not shown). Although these structural alterations may not be related to macrophage or stem cell function, we stopped maintaining the tissue samples longer than 11 days in subsequent experiments. Nevertheless, an 11-day period most likely exceeds the course of macrophage and stem cell responses, since the related regulatory signal transduction, gene transcription, and protein translation were reported to be completed within 48 h [28,29]. Correspondingly, this time frame allows the assessment of potential compounds and their effects on macrophage activation or stem cell differentiation in human skeletal muscle tissue.

We characterized resident macrophages in human skeletal muscle tissue using the restrictive M1 and M2 classification, which may be a limitation of our study. Recent studies indicate that the M1/M2 classification is a technical simplification that may obscure functionally relevant macrophage phenotypes [8,24]. According to this classification, M1 macrophages contribute to a proinflammatory state and M2 macrophages contribute to an anti-inflammatory state via the expression of distinct sets of cytokines. However, blood-derived M2 macrophages were also shown to secrete proinflammatory cytokines [30]. Nevertheless, little is known about macrophage polarization in human skeletal muscle, and a standardized method is still lacking [31]. Indeed, skeletal-muscle-resident macrophages are predominantly studied in rodent injury models that follow the M1/M2 classification [32]. Although application of the skeletal muscle model requires sufficient access to surgical tissue samples, it is a useful tool for the characterization of resident macrophages in the human system.

Previous studies suggested that obesity, diabetes, and insulin resistance increase macrophage accumulation in skeletal muscle without discriminating included adipose or connective tissues [33,34]. In the current study, we determined the number of resident macrophages within the muscle fiber bundles and excluded intramuscular vessels, fat, and connective tissue. Four of the eight included participants suffered from diabetes and/or obesity, which is a limitation of our study (Table 1). However, the results did not confirm the increased accumulation of resident macrophages associated with muscle fibers from donors with diabetes and/or obesity. In particular, the muscle tissues from obese or diabetic participants contained 35% fewer CD80-positive macrophages and 45% fewer CD163-positive macrophages than the other participants (Supplementary Table S1). Therefore, we suggest that diabetes and/or obesity may decrease the number of macrophages that are associated with myofibers specifically. This also may explain the declined muscle regeneration capacity in diabetic or obese older adults. However, both disorders may lead to the increased migration of macrophages into muscle-embedded adipose or connective tissues. An earlier study also found that the skeletal muscle tissue of lean individuals expressed higher levels of the macrophage-specific markers CD163 and CD80 than the skeletal muscle of obese individuals [35]. In another study, the number of resident macrophages in skeletal muscle fibers from obese females with insulin resistance was lower than that in normal-weight females without insulin resistance [36]. Studies of skeletal muscle biopsies from young and older adults found a twofold increase in macrophages in the muscle tissue of healthy 70–81-year-old individuals [37]. Other studies that differentiated between the M1 and M2 subtypes in human skeletal muscle tissue reported a decline in M1 macrophages and an increased anti-inflammatory response with aging [14,38]. Notwithstanding the significance of sex, genetic predisposition, and clinical history, the polarization and function of resident macrophages likely change with aging and therefore need to be specifically addressed in aging muscle tissue. In specific anatomical locations, skeletal muscle fiber type composition and function are different in male and female individuals. However, no sex-related differences were found in the overall structure, tissue, or cellular components of human skeletal muscle tissue [39]. Although our study included mainly female donors, we observed no sex-related differences in muscle tissue structures or the cellular components of skeletal muscle tissue (data not shown). Moreover, we detected no sex-related differences in the viability of macrophages or stem cells in the human muscle tissue model (Supplementary Tables S1–S4). Thus, donors’ sex is unlikely to have affected the viability of this model.

The characterization of stem cells in our study revealed comparable numbers of satellite cells in all participants (Supplementary Table S3). Satellite cells promote muscle tissue regeneration by their proliferation upon activation and expression of a range of myogenic genes, including desmin [40]. In all samples, we found satellite cells in the sarcolemma, where the desmin protein accumulated to the greatest extent. Interestingly, the number of PAX7-positive satellite cells decreased by 50% over 5 days of maintenance in vitro, demonstrating their lack of proliferative capacity. Previous studies suggested that aging may decrease the proliferative activity of satellite cells but not their myogenic differentiation capacity [41]. Interestingly, our data revealed that the significant decrease in PAX7-positive satellite cells was accompanied by a slight increase in MYOD-positive myoblasts. Although the increase in myoblasts was not statistically significant, we assumed that some of the resident satellite cells may have differentiated into myoblasts within five days in vitro. Besides, PAX7 and MYOD proteins may play a role in the regulation of satellite cell differentiation [42]. Analogous to the complexity of macrophage subtypes, the full characterization of stem cells may require additional marker proteins to be identified.

Elevated levels of fatty acids were associated with skeletal muscle loss in numerous animal and human studies. However, little is known about fatty acids’ target cells and their role in regulation of muscle regeneration. Recently, we reported that MYOD-positive primary human skeletal myoblasts were potent producers of chemokines by direct response to distinct species of fatty acids within two days [13]. Here, we found that fatty acids treatment of human skeletal muscle tissue increased the number of resident M1 macrophages and satellite cells within 9 days (Figure 4). Although the levels of cytokines were not examined in our model, it is tempting to speculate that they stimulated the polarization of resident M1 macrophages and the expansion of satellite cells. Moreover, the increased number of M1 macrophages confirmed our initial hypothesis that fatty acids contribute to the inflammatory status in human skeletal muscle tissue. However, future studies need to include more donors to verify the impact of fatty acid species, age, and preconditions in this scenario.

## 5. Conclusions

Human skeletal muscle samples were previously used to capture resident macrophages and stem cells in the static state. Our study presents a new approach that allows the dynamic observation of resident macrophages and stem cells under experimental conditions upon the application of compounds, nutrition, or stimuli. The current embedding technique in agarose medium facilitates the maintenance of viable samples for up to 11 days and the study of resident macrophages and stem cells.

## Figures and Tables

**Table 1:** Characteristics of the study participants.

Participant	Sex	Age (Years)	BMI (kg/m^2^)	Type 2 Diabetes	Source
P1	female	73	26.6	Yes	gluteus medius muscle
P2	female	82	26	Yes	gluteus medius muscle
P3	female	68	>40	Yes	deltoid muscle
P4	female	76	27.3	No	multifidus muscle
P5	female	82	26.1	No	pronator quadratus muscle
P6	male	72	37.5	No	vastus lateralis muscle
P7	female	81	25.6	No	pronator quadratus muscle
P8	female	68	24.8	No	vastus lateralis muscle

## Data Availability

The data presented in this study are available in Supplementary Materials available online at www.mdpi.com.

## Supplementary Materials

The following are available online at https://www.mdpi.com/article/10.3390/biology11060936/s1. Figure S1: The fold change in the sum of PAX7-positive satellite cells and MYOD-positive myoblast over 11 days of maintenance. Table S1: The numbers of CD80-positive and CD163-positive macrophages in the muscle tissue samples from eight participants at day 0. Table S2: The fold change in CD80-positive and CD163-positive macrophages over 11 days. Table S3: The numbers of PAX7-positive satellite cells and MYOD-positive myoblasts in the muscle tissue samples from six participants at day 0. Table S4: The fold change in PAX7-positive satellite cells and MYOD-positive myoblasts over 11 days.
