# Supplementary material for: In Vitro Model of Human Skeletal Muscle Tissue for the Study of Resident Macrophages and Stem Cells
Source: Biology (Basel). 2022 Jun 19;11(6):936. doi: 10.3390/biology11060936 (PMC9219866; doi:10.3390/biology11060936)
Supplement: Supplementary file 1 [file biology-11-00936-s001.zip › biology-1682090-supplementary.pdf]

Figure S1. The fold change in the sum of Pax7-positive satellite cells and MyoD-positive myoblasts over 11 days of maintenance.

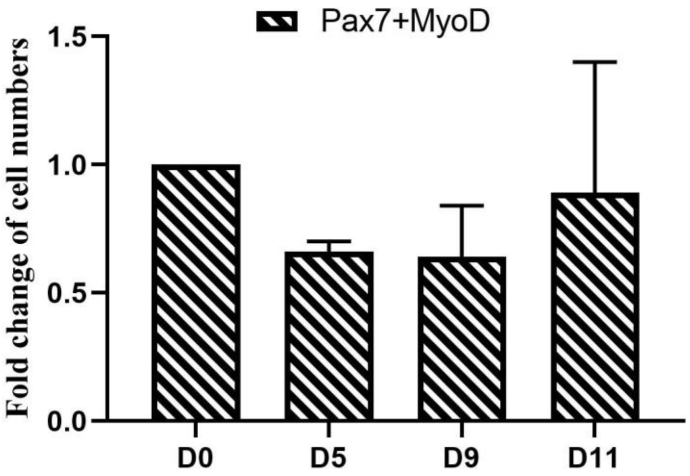

Table S1. The numbers of CD80-positive and CD163-positive macrophages in the muscle tissue samples from eight participants at day 0.

participants	CD80				CD163			
	field 1	field 2	Mean	SD	field 1	field 2	Mean	SD
P1	30	38	34.0	4.0	18	18	18.0	0.0
P2	12	18	15.0	3.0	13	10	11.5	1.5
P3	24	17	20.5	3.5	18	13	15.5	2.5
P4	13	11	12.0	1.0	30	34	32.0	2.0
P5	41	26	33.5	7.5	25	32	28.5	3.5
P6	35	41	38.0	3.0	35	46	40.5	5.5
P7	57	49	53.0	4.0	40	37	38.5	1.5
P8	46	47	46.5	0.5	25	27	26.0	1.0

Note: 8 participants samples after surgery (day 0) immediately was analyzed the cell numbers of skeletal muscle M1 and M2 marked by CD80 and CD163 respectively. In each sample, the mean number of positive cells was obtained from at least two randomly selected fields of 0.24 mm^2 within cross-sectional areas of muscle fibers. The fields of adipose tissue, glands or vessels were strictly excluded.

Table S2. The fold change in CD80-positive and CD163-positive macrophages over 11 days.

days	CD80			CD163		
	Mean	SD	*P* value	Mean	SD	*P* value
D0	1.00	0.00		1.00	0.00	
D5	1.06	0.27	0.023	0.96	0.19	0.064
D9	1.00	0.24	0.069	0.92	0.16	0.023
D11	1.04	0.35	0.043	0.83	0.17	0.00115

Note: D0 was set to one for every single participant and for the mean change at D0, accordingly. The number of CD80 or CD163 at D5, D9, D11 were compared to their numbers at D0 to obtain a relative fold change and the corresponding mean value of relative fold changes. Statistical significance was calculated using T-test and p value ≤0.01 were considered as statistically significant.

Table S3. The numbers of Pax7-positive satellite cells and MyoD-positive myoblasts in the muscle tissue samples from six participants at day 0.

participants	Pax7				MyoD			
	field 1	field 2	Mean	SD	field 1	field 2	Mean	SD
P1	24	28	26.0	2.0	25	17	21	4
P2	13	10	11.5	1.5	15	20	17.5	2.5
P3	21	27	24.0	3.0	18	10	14	4
P5	8	10	9.0	1.0	5	12	8.5	3.5
P6	21	18	19.5	1.5	16	14	15	1
P7	23	11	17.0	6.0	24	15	19.5	4.5

Note: 6 participants samples after surgery (day 0) immediately was analyzed the cell numbers of skeletal muscle satellites and myoblasts marked by Pax7 and MyoD respectively. In each sample, the mean number of positive cells was obtained from at least two randomly selected fields of 0.24 mm^2 within cross-sectional areas of muscle fibers. The fields of adipose tissue, glands or vessels were strictly excluded.

Table S4. The fold change in Pax7-positive satellite cells and MyoD-positive myoblasts over 11 days.

days	Pax7			MyoD		
	Mean	SD	*P* value	Mean	SD	*P* value
D0	1.00	0.00		1.00	0.00	
D5	0.52	0.19	0.019	0.82	0.16	0.018
D9	0.45	0.17	0.009	0.83	0.24	0.092
D11	0.60	0.30	0.052	1.24	0.73	0.058

Note: D0 was set to one for every single participant and for the mean change at D0, accordingly. The number of Pax7 or MyoD at D5, D9, D11 were compared to their numbers at D0 to obtain a relative fold change and the corresponding mean value of relative fold changes. Statistical significance was calculated using T-test and p value ≤ 0.01 were considered as statistically significant.
